# Medical Education—Lengthened College Terms—Biennial, Instead of Annual Commencements for Conferring Degrees

**Published:** 1856-03

**Authors:** 


### EDITORIAL.

*Medical Education—Lengthened College Terms—Biennial, instead of Annual Commencements for Conferring Degrees.*

Some months since, we very briefly noticed the proposition made by Dr. Stille, of Philadelphia, in the last annual meeting of the American Medical Association, to extend each of the courses usually given in our Medical Colleges, so as to make them occupy two annual sessions instead of one. We then stated that the proposition was referred to a Special Committee, of which Dr. Stille was Chairman. Very recently we have received a circular from said Committee, setting forth more fully the nature of the changes proposed, and the reasons in favor of their adoption. The views contained in the Circular may be stated briefly as follows:—

1st. To make the College Cirriculum consist of eight distinct courses of instruction, namely:

"Special, Regional, and General Anatomy."

"Inorganic, Organic, and Pharmaceutical Chemistry, and Toxicology."

"Medical Botany, Materia Medica, General Therapeutics."

"General Pathology, Morbid Anatomy, Practice of Medicine."

"General and Human Physiology, Hygiene, Medical Jurisprudence."

"General Surgical Pathology, Mechanical, Operative, and Medicinal Surgery."

"Obstetrics, Diseases of Women, Diseases of Children."

"Hospital Clinical Medicine and Surgery."

2d. To make the course of Lectures on each of the foregoing departments extend through two annual College terms, instead of having each annual course, to a great extent at least, a *repetition* of the one that preceded it.

3d. To make the Commencement for Conferring Degrees biennial instead of annual, as at present. This necessarily results from the adoption of the second proposition. For if it require two annual college terms to complete the several courses of Lectures, each student would necessarily be required to enter the same college for *two terms,* and a graduating class could be formed only at the end of each second term.

The important objects or benefits to be gained by the proposed change, as set forth in the circular of the Committee, are, *first*, to render the college instruction of the student *developmental* and *progressive* from the beginning to the end of his college course, instead of annually *repetitional*, as at present. Second, to afford opportunity for including in the several departments, instruction on important topics which are now either wholly omitted or but imperfectly presented. Such is the plan for improving our system of college instruction, proposed by the Committee, and such the benefits which it is claimed would arise from its practical adoption. That the time has fully come when the *curriculum* of medical college instruction in our country should be thoroughly remodeled, and placed on such a basis that the student can receive instruction in a limited number of branches each year of his pupilage, beginning with the elementary and ending with the *applied* or practical, there can be no doubt in the reflecting and unbiased mind. But does the plan proposed by the Committee present such a basis? By no means. On the contrary, it proposes to have the student *commence* his college instruction, with *eight* distinct departments or courses of Lectures, (including the whole fields of Medical Sciences,) and pursue them all halfway through the first annual term, and return to complete them the second. This would be "progressive" instruction as distinguished from "repetitional;" but it would not be "*developmental*," as claimed by the Committee. To develop a science, or a connected chain of sciences, we must begin with the elementary parts—we must master first those topics which legitimately and necessarily furnish the basis and key to the subsequent attainments. To acquire a knowedge of the Science and Art of Medicine by a true system of *development*, the student must master Anatomy, General and Descriptive; Chemistry, Organic and Inorganic; Botany and Materia Medica; and Physiology, Human and Comparative; *before* he proceeds to the study of Morbid Anatomy; Toxicology; and General Pathology, Medical and Surgical. And all these must precede the full consideration of Practical Medicine, Operative Surgery, and Obstetrics. Instead of this, however, the plan of the Committee requires the student to commence his first college term with Lectures on the elements of Anatomy, Chemistry, Materia Medica, Physiology, General Pathology, Surgical Pathology, Obstetrics, and Clinical Practice; thereby crowding daily quite as many topics upon the mind of the student as under the present system, and quite as perfectly destroying all power to pass, by a natural and consecutive order, from the acquisition of one series of branches to the next and most dependent. Hence the plan proposed is scarcely less objectionable than the one already in operation in the schools; while its adoption would subject the student to the very great inconvenience of being compelled *always* to enter the medical college for two *consecutive terms*, or be deprived of the opportunity to complete what he had begun until two whole years had intervened, and he had reached the *fourth* college term. The Committee, on the second page of the circular before us, seem to convey the idea that among all the plans heretofore proposed for improving our system of medical college instruction, none have had for their object the avoidance of "the repetitional system," and "the substitution of a system of *development*," therefor. This is certainly a mistake. So long since as the year 1850, we wrote and published a little volume, entitled "A History of Medical Education and Institutions in the United States," with a Chapter on the "Condition and Wants of the Profession."

If Dr. Stille and his colleagues on the Committee will take the trouble to look over the pages of that work, they will find the proposition to exchange our present *repetitional* college system for one of true *development*, not merely hinted at, but most clearly and emphatically urged upon the attention of the reader.

To aid the memory of our readers, we quote as follows, commencing on page 218 of the History referred to:—

"If we would ever raise the medical profession to that condition, in regard to learning and practical skill, which justice to itself and the community imperiously require, two things must be done:—

1st, All those medical colleges that are so located as to afford their students the necessary facilities for the study of anatomy, healthy and morbid, and the free access to well-regulated hospitals, must not only double the length of time during which their lectures continue, but such time must be suitably divided into minor terms. Thus, if the colleagues in all our large cities would continue their course of instruction through nine or ten months, dividing such time into three sections, and assigning four distinct branches to each section, thereby occupying four hours each day in the lecture room, instead of six, and requiring the student to spend the remaining two hours in clinical study at the hospital, including dead-house observations in morbid anatomy, and personal practice in physical diagnosis, they would not only present the requisite facilities for obtaining the right kind of medical knowledge, but would also give the student a chance to avail himself of those facilities. The different branches should be so grouped in the three sections, that all students studying three years would find it profitable to take tickets, and attend on one section only each year of his study.

With such a college arrangement, the student would be enabled to take up four branches each successive year of his study, and by confining his attention to these, both during his attendance on the college and in the interval, instead of skimming each year over the whole field of medical sciences, he would make himself thoroughly acquainted with each in its turn, and, at the same time, acquire a mental discipline, far superior to that obtained under the present arrangement.

At present, first course students, many of whom have scarcely studied long enough to learn the number of bones in the human skeleton, are compelled to pay for tickets and listen to lectures on practical medicine, surgery, and midwifery, before they have the slightest knowledge, either of the organs diseased, the remedies recommended, or the anatomical structures operated on. In a word, they are set to laying the foundation, building the superstructure, and putting in the furniture, all at the same time—a task as difficult as it is absurd. But let the whole annual session be lengthened and divided, in the manner proposed, and students, in the first year of study, can take the tickets for that section adapted to their period of progress, and concentrate all their attention on a thorough mastery of anatomy, and the other more elementary branches; those in the second year would take the tickets of another section, adapted to their stage of progress, and spend their time between the lectures in paying special attention to morbid anatomy and pathology, illustrated by post-mortem examinations; while those in the third or last year, having thus laid a systematic foundation, would take the section embracing instruction in the more purely practical branches, and be able to devote all their time out of the lecture room to genuine clinical medicine and surgery at the bedside. Such an arrangement would require the student to attend the college no longer in any one year than he does now, would compel him to attend no more branches at one time than he could profit by to the greatest advantage, and yet would give him ten months of college instruction during his period of study, instead of eight, which is now required."

In our estimation, this plan has many advantages over that proposed by the Committee in their circular. It calls for a greater division and more perfect grouping of topics, while it embraces an equally extensive field of instruction, without necessitating the inconvenience of *bi-ennial* instead of annual *commencements*, and the still greater inconvenience of compelling the student, in all cases, to attend the same college two consecutive terms, or have all his courses of instruction broken and incomplete. The plan of college instruction proposed by us, also more fully meets the wants of the profession and the deficiencies of the present system. What are these wants?

So far as medical colleges are concerned, they are first, the presentation of a less number of topics to the mind of the student each day in the lecture room, and the allowance of more time for dissection, clinical instruction, and reflection. Second, such a division and extension of the annual college terms as will enable students, attending their first course, to attend lectures on such branches only as are adapted to the period of their advancement; while those listening to a second course should have another series of branches, and so on in consecutive order until fitted for graduation. Examinations should be made at the end of each division of the annual college term, and no student should be allowed to proceed in attendance on a second or third division of the term until he had borne a satisfactory examination on all the branches included in the preceding division. The adoption of any system by our colleges which would supply these wants, or more properly, accomplish these objects, would speedily reduce the study of medicine to a strictly methodical, progressive, and *developmental* process. It would compel students every where to commence certain branches and master them, before they could proceed to others. It would completely destroy a most pernicious practice, long prevalent, especially in the West; which consists in the desultory study of medicine from eighteen months to two years, the attendance on three or four months of lectures, during which they *skim* over the whole field, and then forthwith engage in practise, without presenting themselves either for an examination or a degree. The adoption of such a system would also enable the faculty of each college to include in its curriculum of instruction, for *each collegiate year*, all the branches proposed by the Committee, of which Dr. Stille is chairman, and discuss them as fully as he proposes to do in two years.

The two great and paramount evils of our present system of medical college instruction, are, first, the inadequate time allowed in a session of four or five months, to present the various branches of medical science in that degree of detail which is desirable; and second, the grouping together of students in all stages of progress in their study of medicine, and lecturing to them daily on six or seven different topics, by which the novice who has not yet studied six months listens, on the same day, to instruction in the elements of Anatomy and Physiology, and in the most complex theories and phenomena of Practical Medicine and Surgery. In the first serious efforts to improve our system of medical education, resulting from the organization of the National Association, it was proposed to remedy these evils by lengthening the annual college terms to six months. But it is easy to perceive that, if in adopting a *six* months' term, the number of lectures are reduced so as to admit of adequate attention to dissection, physical diagnosis, and clinical instruction, the diminished number per day would fully offset the increased length of term, and hence there would be no positive gain in the extent or fulness of the several courses; therefore, the first evil would remain with but little amelioration. On the other hand, if from a desire to extend the several courses, the same number of daily lectures are continued, the second evil remains in full force, being only aggravated by protraction through a longer period of time. The plan proposed by Dr. Stille remedies, in part at least, the *first* great evil named, by really extending the several courses of lectures to a greater length and completeness; but it leaves the second, and *greater* evil, entirely unmodified. We should be compelled, therefore, to object decidedly to its adoption. If we are to have any remodeling of the general curriculum of medical college instruction in our country, it should be done with a full comprehension of the evils to be obviated and the benefits to be gained. After years of reflection, and some personal experience in the business of teaching, we are more than ever satisfied that the plan, set forth in the extract quoted above, presents the basis of a system more perfectly adapted to the wants of the profession than any other which can be devised. It provides a length of college term sufficient to permit the presentation of every important branch of medicine as fully as is desirable; and yet allows time, out of the college lecture room, for dissections and clinical observations. It causes the lectures on the several branches to be given in a consecutive order, according to their natural relations and dependencies, thereby causing the whole course of study to be one of method and true progressive development. Without increasing the pecuniary burdens of the student, it enables him to attend each year, that division of the annual college term *only*, which includes instruction on those branches that belong to his particular period of advancement in his pupilage. By adopting such a lengthened college term, subdivided into sections, other advantages would be gained of much importance.

There are certain portions of the instruction which should be given in connection with all our colleges, that can only be given to a limited number at one time. We allude to instruction in minute anatomy, whether healthy or morbid, by means of the microscope; to individual training in physical diagnosis, which is the only method by which the student can acquire any adequate degree of knowledge in that important department; and to true hospital clinical instruction at the *bedside* of the sick. Only a very limited number can receive instruction in either of these important departments at one time; and hence it is almost impossible to make any arrangements by which a class of 400 or 500 students, in all periods of advancement in study, can receive that personal attention in these departments which is absolutely necessary for their proper qualification to practice the healing art. If, however, we extend and subdivide the annual college term, in such a manner that the 400 or 500 can be divided, according to the period of their studies, between the several sections of the collegiate year, there would be in attendance on no one section or subdivision of the term, so large a number as to prevent their full and thorough instruction in every important department; and yet neither the aggregate number of students in attendance on the college during the year, nor the pecuniary receipts would be diminished. Neither would the adoption of such a system necessarily require the support of a larger number of professors in connection with each college; for the duties devolving on the several chairs, being discharged during successive sections of the annual college term, there would be no inconvenience in having the same teacher fill a chair in two successive sections of the college term. On the contrary, such a course, in reference to some of the departments, would doubtless secure the highest degree of uniformity and completeness in the courses of instruction given.

That the system of medical college instruction in this country needs revision, is obvious to every intelligent and reflecting member of the profession; and we most sincerely wish that a conference of delegates from the several colleges could be held, to give the whole subject a candid, thorough, and liberal consideration.

